# Proteases and Their Inhibitors in Chronic Obstructive Pulmonary Disease

**DOI:** 10.3390/jcm7090244

**Published:** 2018-08-28

**Authors:** Tapan Dey, Jatin Kalita, Sinéad Weldon, Clifford C. Taggart

**Affiliations:** 1 Biological Sciences and Technology Division, CSIR-North East Institute of Science and Technology, Jorhat 785006, Assam, India; rs_tapandey@dibru.ac.in (T.D.); kalitajk74@gmail.com (J.K.); 2 Centre for Biotechnology and Bioinformatics, Dibrugarh University, Dibrugarh 786004, Assam, India; 3 Airway Innate Immunity Research Group, Centre for Experimental Medicine, School of Medicine, Dentistry and Biomedical Sciences, Queen’s University Belfast, Belfast, Northern Ireland BT9 7BL, UK; s.weldon@qub.ac.uk

**Keywords:** chronic obstructive pulmonary disease, protease, antiprotease

## Abstract

In the context of respiratory disease, chronic obstructive pulmonary disease (COPD) is the leading cause of mortality worldwide. Despite much development in the area of drug development, currently there are no effective medicines available for the treatment of this disease. An imbalance in the protease: Antiprotease ratio in the COPD lung remains an important aspect of COPD pathophysiology and several studies have shown the efficacy of antiprotease therapy in both in vitro and in vivo COPD models. However more in-depth studies will be required to validate the efficacy of lead drug molecules targeting these proteases. This review discusses the current status of protease-directed drugs used for treating COPD and explores the future prospects of utilizing the potential of antiprotease-based therapeutics as a treatment for this disease.

## 1. Introduction

Chronic obstructive pulmonary disease (COPD) is considered to be one of the major diseases of modern times. With a continuous rise in pollution across the globe, combined with continued cigarette smoking in both developing and developed countries, COPD is set to become the third leading cause of death by 2030 [1,2]. Despite major efforts to find a treatment for COPD, effective therapeutic strategies remain elusive [3,4,5,6,7]. COPD is a progressive lower respiratory tract disorder encompassing chronic bronchitis and emphysema. Chronic bronchitis is caused by increased secretion of mucus from differentiating goblet cells leading to a thicker mucus layer lining the airways [8]. Emphysema is caused by the destruction of the terminal bronchioles leading to decreased gas exchange in the lower airways [9]. Both diseases result in decreased pulmonary function and quality of life. Cigarette smoking is considered the primary cause of COPD, although only 15–20% of smokers are thought to develop COPD. This fact can be rationalized by the fact that around 90% of COPD cases are either ex-smokers or current smokers [10,11]. Moreover, around 1–5% of COPD cases have an underlying genetic component caused by a deficiency of the serum glycoprotein α-1 antitrypsin (A1AT) [12,13]. A1AT is the primary serine antiprotease responsible for protecting the lungs against the actions of neutrophil-derived serine proteases.

COPD is also considered to be an age-related disorder [14,15]. Therefore, with an increase in the worldwide aging population, the number of patients being diagnosed with COPD is also on the increase. Presently, bronchodilators are the mainstay treatment for the management of COPD but fall short of overall effectiveness [16,17,18]. In addition to environmental insults such as cigarette smoke, bacterial and viral pathogens may also play a major role in the development of COPD and contribute to the rise in exacerbation frequency among the COPD population [19,20,21]. Given the increasing healthcare and societal burden associated with the increase in COPD, a number of therapeutic programmes are ongoing to develop strategies for the treatment of COPD [22,23,24,25,26,27,28,29,30]. As it will be beyond the scope of this review to discuss all of the ongoing therapeutic programmes, we will focus on the current status of antiprotease therapy against COPD.

## 2. Proteases Involved in COPD Pathophysiology

The protease: Antiprotease imbalance is considered to be one of the core physiological mechanisms involved in the pathogenesis of COPD [31]. One of the major genetic causes of COPD is A1AT deficiency [32]. A1AT is a serine protease inhibitor which regulates the neutrophilic chemotaxis involving both CXCR1 and FcγRIIIb signaling [33]. In addition, A1AT has also been shown to regulate the levels of cathepsin B and metalloproteinase-2 (MMP2) in A1AT deficient patients treated with A1AT augmentation therapy [34].

There are four different types of proteases namely serine, cysteine, aspartic, and MMPs which are thought to be involved in the pathogenesis of COPD. Among serine proteases, specifically, neutrophil elastase (NE), dipeptidyl peptidase 4, cathepsin G, proteinase 3, cathepsin C, mast cell-derived tryptase and chymase are found to be associated with the severity of COPD [35,36,37,38,39,40]. The metal-activated proteinases including MMP-2, MMP-8, MMP-9, MMP-12, and MMP-13 are found to be highly expressed in both in vivo models and clinical samples [41,42,43,44,45,46]. The cysteine proteases including caspase-1, caspase-3, caspase-7, caspase-8, caspase-9, caspase-11, cathepsin K, and cathepsin S have also been shown to be up-regulated in COPD patients [47,48,49,50,51,52,53,54,55]. Finally, elevated levels of the aspartic proteases cathepsin D and cathepsin E have been demonstrated in COPD patient tissue and various COPD models [56,57,58,59].

## 3. The Role of Serine Proteases and Their Inhibitors in COPD

### 3.1. Neutrophil Elastase (NE)

Neutrophil Elastase (NE) is the primary enzyme present in azurophil granules in the neutrophil cytoplasm and is thought to play a role in the proteolytic breakdown of phagocytosed molecules. Neutrophils are the first cell type to arrive in the lung following stimulation by microbial pathogens, smoking, and various other environmental exposures [60]. However, unopposed NE activity in the lung may lead to lung parenchyma destruction and subsequent increased production of inflammatory mediators [61]. A1AT is considered to be the primary regulator of NE activity. In normal lungs, A1AT accounts for around 90% of anti-NE activity at the lower respiratory tract providing protection to the underlying connective tissues [62]. Elevated serum levels of NE have been found to be associated with COPD severity [63]. In addition, higher NE concentrations exist in saliva and exhaled breath condensate of COPD patients [64,65]. Therefore several previous studies have focused on the inhibition of NE as a treatment strategy for COPD.

**Figure 1:**
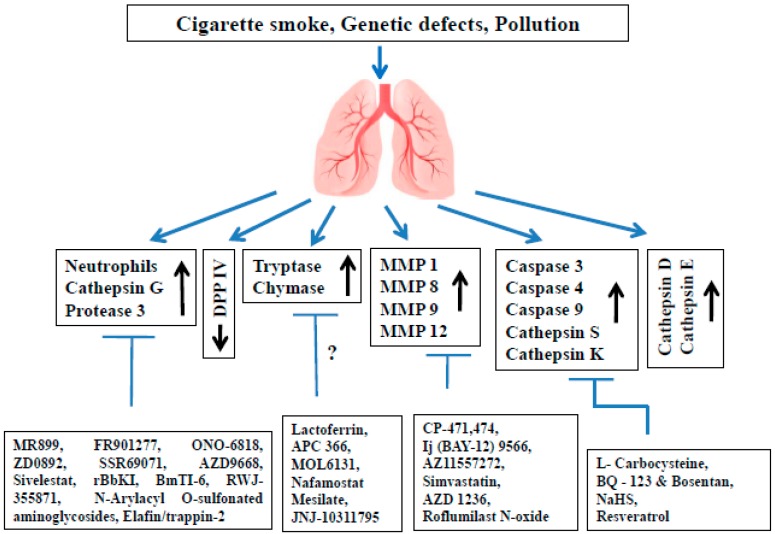
Present status of different protease inhibitors tested against COPD models. COPD: Chronic obstructive pulmonary disease; DPP IV: Dipeptidyl peptidase IV; MMP: Matrix metalloproteinase.

**Figure 2:**
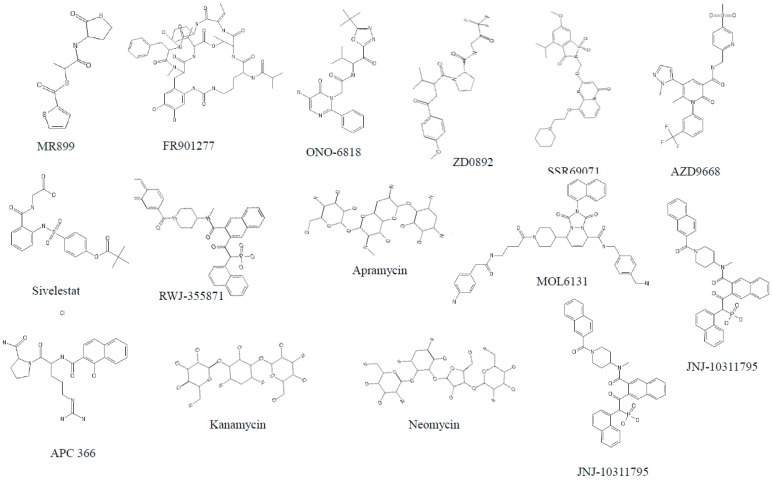
Structure of different serine protease inhibitors tested against COPD models.

MR899 was the first NE inhibitor used in clinical trials for COPD (Figure 1 and Figure 2) [66]. MR899 is a cyclic thiol compound derived from homocysteine lactone and thiolactic acid. It was found to be a competitive and reversible NE inhibitor. Oral administration of MR899 at a dose of 500 mg twice a day for 4 weeks was tested to check its efficacy in reducing levels of lung destruction markers. In this study, urinary levels of desmosine and plasma elastin-derived peptides thought to be derived from NE activity were measured. Interestingly, MR899 was found to be effective only in those COPD patients who had only recently been diagnosed with COPD and who had less established disease.

FR901277 is a cyclic peptide lactone isolated from the fermentation broth of *Streptomyces resistomycificus*. FR901277 was shown to be effective in reducing porcine pancreatic elastase (PPE)-induced emphysema in hamsters (Figure 1 and Figure 2). The median effective dose at around 8 mg/kg body weight by intratracheal instillation effectively inhibited the increase observed in lung compliance and vital capacity of the lungs after 2 weeks of PPE treatment. However, despite these promising studies follow-up studies using FR901277 have not been carried out. ONO-6818 was found to effectively reduce the lung hemorrhage and neutrophil accumulation associated with NE-induced rat emphysema model (Figure 1 and Figure 2) [67]. Oral pre-administration of ONO-6818 at a dose of 100 mg/kg was found to reduce increased hemoglobin concentration as well as neutrophil count and myeloperoxidase activity in bronchoalveolar lavage (BAL) fluid within 6 h of human NE instillation. In addition, histopathology studies demonstrated a decrease in emphysematous changes in the ONO-6818 treated group. Furthermore, ONO-6818 was shown to reduce NE-induced increases in lung compliance and mean linear intercept (L_m_) in the rat model. However, despite these promising results ONO-6818 was later shown to have a deleterious effect on liver function.

In another study, the specific NE inhibitor, ZD0892, was shown to have a profound effect in both the acute and chronic phase emphysema guinea pig models (Figure 1 and Figure 2) [68]. Oral administration of ZD0892 at a dose range of 3–30 mg/kg resulted in lower total neutrophil cell counts in BAL fluid in a dose-dependent manner. Moreover, it lowered the levels of desmosine and hydroxyproline in BAL fluid. In addition, the expression of inflammatory mediators such as macrophage inflammatory protein 2 (MIP-2), monocyte chemoattractant protein 1 (MCP-1) and tumor necrosis factor-α (TNF-α) were also found to be reduced upon ZD0892 administration. More importantly, ZD0892 was also found to effectively reduce inflammation in chronic smoke exposed guinea pigs. Interestingly SSR69071, a saccharide derivative was found to be more potent than earlier tested NE inhibitors [69]. SSR69071, when orally pre-administrated at a dose range of 0.3 to 30 mg/kg body weight before elastase instillation, was shown to effectively reduce elastase-induced lung hemorrhage in mice. SSR69071 was also shown to decrease lung hemorrhage and lung injury.

Preclinical studies with AZD9668, an orally available NE inhibitor has found it to be effective against both human NE- and cigarette smoke-induced emphysema models (Figure 1 and Figure 2) [70]. Affinity studies with AZD9668 have shown it to bind NE more rapidly compared to ONO-6818. Moreover, it showed more specificity for NE compared to other NE inhibitors such as ONO-6818 and sivelestat. In an acute NE instilled model, AZD9668 was shown to effectively reduce BAL hemoglobin level at a dose of >1.5 mg/kg and BAL hydroxyproline and desmosine levels at a dose of 2.5 mg/kg and 10 mg/kg, respectively. In a chronic smoke-induced emphysema mouse model, AZD9668 was shown to effectively reduce BAL neutrophil levels at a dose of 6 mg/kg and BAL IL-1β level at a dose of 1 mg/kg body weight. Moreover, AZD9668 was shown to completely prevent airspace enlargement (emphysema) and small airway remodeling in chronic models. Although AZD9668 was shown to be effective in preclinical models, it was shown to be ineffective in clinical trials of COPD. In a randomized, placebo-controlled phase IIb trial, three months treatment with AZD9668 improved neither the lung function nor the sign and symptoms associated with COPD patients with a history of budesonide/formoterol therapy [71]. In another clinical trial in COPD patients, AZD9668 at a dose of 60 mg twice a day did not reduce inflammation or lung damage when applied in combination with tiotropium [72].

In addition to synthetic protease inhibitors, plant-derived protease inhibitors have been evaluated. *Bauhinia bauhinioides L.*, a plant from the Caesalpinioideae sub-family has been shown to secrete many protease inhibitors. Among them, *Bauhinia bauhinioides* Kallikrein proteinase Inhibitor [73] (rBbKI) and *Bauhinia bauhinioide scruzipain* inhibitor [74] (BbCI) have been found to effectively ameliorate elastase-induced emphysema (Figure 1). In a model of elastase-induced emphysema model, rBbKI was shown to effectively reduce elastase-induced inflammation and extracellular matrix remodeling. Moreover, rBbKI reduced the number of BAL cells and inflammatory markers including TNF-α, lung remodeling markers (MMP-9, MMP-12, and TIMP-1), and oxidative stress markers (eNOS and iNOS) markers in respiratory airways and alveolar walls. In addition, rBbKI diminished the increase in lung mechanical stress parameters such as respiratory system elastance, respiratory system resistance, airway resistance, lung tissue elastance and lung tissue damping. BbCI was also shown to effectively ameliorate lung inflammation and extracellular lung remodeling at a dose of 2 mg/kg.

Recently, the potency of an arthropod-derived serine protease inhibitor in the elastase-induced emphysema model was evaluated [75]. In the study, the authors employed BmTI-6, a Kunitz-type serine protease inhibitor to test its efficacy against elastase-induced emphysema model (Figure 1). The lung L_m_ was found to be reduced in the recombinant BmTI-6-D1 *(Domain 1) treated group. In addition, the BmTI-6-D1 instillation reduced the respiratory mechanics and the macrophages, neutrophil and lymphocyte count in BAL fluid. Moreover, it increased the volume proportion of collagen and elastic fibers and decreased NE activity compared to the elastase only treated group.

### 3.2. Cathepsin G (cat G)

Cathepsin G (cat G) is one of the three major serine proteases secreted by the azurophilic granules of neutrophils [76]. In addition to its antibacterial activity, cat G plays a role in innate immunity, chemoattraction and extracellular matrix degradation [77,78]. Cat G was found to protect against *Streptococcus pneumoniae*-induced lung damage [79]. In contrast, genetic knockdown of cat G has also been found to protect lung tissue destruction from long-term exposure of cigarette smoke [37]. Moreover, increased expression of cat G can lead to alveolar wall destruction and abnormal secretion of mucus from the airway serous cells in COPD patients [80,81,82].

Garavilla et al. described the cat G inhibitory activity of RWJ-355871 in lipopolysaccharide (LPS)-induced acute inflammation model (Figure 1 and Figure 2) [83]. Upon aerosolized instillation of RWJ-355871, levels of exhaled nitric oxide were reduced by 20–37% in this model. In addition, neutrophil, lymphocyte, monocyte, eosinophil, and basophil counts in BAL among in the RWJ-355871 treated group were found to be significantly reduced compared to control groups. In another set of experiments, by the same group, RWJ-355781 treatment did not reduce the total cell count in the BAL of treated animals, however, it reduced the neutrophilic load by 66% in the smoke-induced inflammation model [84]. In addition, RWJ-355781 instillation reduced the levels of keratinocyte-derived chemokine (KC), a murine homolog of IL-8, in a smoke-induced acute inflammation model. These studies demonstrate the efficacy of RWJ-355781 in acute inflammation models. However, in-depth studies in chronic lung models are required in order to establish the potential of RWJ-35578 for the treatment of chronic lung inflammation associated with COPD. Recent studies by Cracian et al. demonstrated the potential of N-Arylacyl O-sulfonated aminoglycosides for cat G inhibition in in vitro models [85]. The aminoglycoside derivatives of neomycin, kanamycin, and apramycin showed significant inhibition of cat G at IC_50_ doses ranging from 0.42 to 209 µM. However, further in vivo studies will be required to determine the therapeutic potential of these aminoglycosides for the treatment of COPD.

### 3.3. Proteinase 3 (PR3)

Proteinase 3 (PR3) is the most abundant serine protease present in the azurophilic granules of neutrophils [86] and is mostly active in the immune response to infection and is an autoantigen in Wegeners’ disease [87]. In addition, it possesses antibacterial activity against a host of pathogens such as *Pseudomonas aeruginosa*, *Staphylococcus aureus*, *Aspergillus fumigatus*, and *Candida albicans* [88]. As degranulation-associated neutrophilic inflammation was found to play a major role in COPD pathophysiology, PR3 has also received attention with regard to its potential role in inflammation. PR3 also takes part in various pro-inflammatory responses such as activation of TNF-α and IL-1β [89]. The PR3 concentration as well its activity was found to be up-regulated during exacerbations in COPD in contrast to the levels found in stable COPD patients [90]. In addition, mice deficient in PR3 were significantly protected from lung tissue destruction after long-term cigarette smoke exposure for 6 months [37]. These studies suggest a role for PR3 in COPD pathophysiology.

Elafin/trappin-2, an innate serine protease inhibitor primarily secreted by epithelial cells, was found to regulate PR3 activity (Figure 1 and Figure 2). In the PPE-induced emphysema model, trappin-2 reduced lung neutrophil accumulation within 24 h of intranasal administration [91]. An engineered trappin-2, trappin-2 A62L, decreased PR3 induced pro-inflammatory cytokines such as IL-6 and IL-8 by lung cells [92]. In addition, the engineered NE-resistant variants, GG- and QQ-elafin, showed prominent anti-inflammatory activity compared to WT-elafin. The GG-elafin variant was shown to reduce inflammation in both LPS challenged in vitro and acute in vivo lung inflammation models [93]. In a yet another study of elastase-induced emphysema, WT-elafin was shown to protect against lung destruction and prevent neutrophil alveolitis [94].

In addition to innate inhibitors, several types of synthetic PR3 inhibitors have been evaluated for their efficacy. Among them, kanamycin derived N-arylacyl O-sulfonated aminoglycoside, KanCbz, has been shown to have the most potent IC_50_ (16 µM) against PR3 compared to other tested derivatives (Figure 1 and Figure 2) [85]. Though a large number of studies have shown anti-inflammatory properties of elafin, there was no clinical evaluation of this inhibitor in COPD. Therefore, clinical trials with elafin or its functional variants may be an interesting future treatment option for COPD.

### 3.4. Dipeptidyl Peptidase IV (DPP IV)

DPP IV, commonly known as cluster of differentiation 26 (CD26), is a cell surface serine protease which primarily cleaves X-proline or X-alanine dipeptides from the N-terminus of polypeptides [78]. DPP IV is expressed both as a type II transmembrane protein and in soluble form [95,96]. Pertaining to its ubiquitous in nature, it is also found in the respiratory tract in the lung parenchyma (type I and II cells), interstitium and in alveolar macrophages and mononuclear lymphoid cells [97].

Decreased serum levels of DPP IV were found to be associated with COPD pathogenesis, independent of age and smoking history [36,98]. More importantly, elevated levels of DPP IV was also associated with acute exacerbation in COPD patients [36]. Interestingly, the lung tissue of smokers and end-stage COPD patients were demonstrated to have higher expression of DPP IV than non-smoker tissue [99]. Moreover, immunostaining studies on airway epithelia, pleural mesothelia, and alveolar macrophages of COPD patients were shown to have enhanced expression of DPP IV [97]. Owing to its neutrophil chemorepellant nature, DPP IV may serve as an augmentation therapy for COPD. Several lines of evidence have shown that soluble recombinant DPP IV may have important anti-inflammatory effects [100,101,102]. Herlihy et al. showed that recombinant human DPPIV treatment (2 µg/mL) reduced neutrophil infiltration in a type II collagen-induced lung inflammation model [100]. In addition, DPP IV was also found to regulate C-X-C motif chemokine 12 (CXCL12), which primarily activates the inflammatory cascade stimulated by inflammatory stimuli such as LPS [101]. The release of DPP IV from the cell membrane into the circulation may be important in COPD pathogenesis. MMPs were found to be associated with the release of DPP IV from the cell membrane [103]. As elevated levels of a number of MMPs were also found to be associated with COPD, this may represent a mechanism to explain elevated levels of DPP IV in COPD.

### 3.5. Tryptases

Tryptases are tetrameric serine proteases secreted by mast cells [104]. They are the most abundant form of serine proteases secreted by mast cells during anaphylactic shock [105]. There are two types of tryptases, namely, α-tryptase and β-tryptase [106]. In addition to its active role in allergic reactions, tryptases have also been found to be associated with smoking-related chronic lung diseases. An increase in tryptase levels was found in the BAL of smokers [107]. Several studies have shown apositive correlation between peripheral airway tryptase positive cells and lung function (FEV_1_/VC) in patients with COPD indicating a possible role for tryptases in this disease [40,108]. In contrast, tryptase levels were found to be lower in subepithelial layer of central airways of COPD patients and not found to correlate with lung function [109]. In addition to its high cell count, the level of tryptase activity was found to be elevated (3.4 times) in patients with severe COPD compared to mild COPD patients [110]. Although a number of tryptase inhibitors such as lactoferrin, APC 366, MOL 6131, and nafamostat mesilate have been used to study the underlying signaling mechanisms in allergic induced airway disease models [111,112,113,114], a study pertaining to COPD pathophysiology is yet to be carried out (Figure 1). Thus, pre-clinical studies to evaluate tryptase inhibitors in COPD models maybe useful to delineate further a role for this protease in COPD.

### 3.6. Chymases

Chymases are serine proteases secreted by the mast cells and possess cathepsin G-like specificity [115]. The primary function of chymases is the conversion of angiotensin-I to angiotensin-II. Excessive leakage of chymase due to higher mast cell degranulation by different stimulants leads to cellular matrix degradation, activation of TGF-β/Smad signaling, conversion of active MMPs from their zymogen form, and activation of several interleukins (such as IL-1β, IL-18, etc.) and endothelins [116,117,118]. There are basically two types of chymases: α-chymases and β-chymases. The chymases present in humans are α-class whereas rodents possess β-chymases in addition to α-chymases [119]. In addition to their role in vascular diseases, chymases were also found to be associated with lung diseases such as pulmonary fibrosis, pulmonary arterial hypertension (PAH), asthma, and COPD [116,120,121,122]. Several investigators have shown a higher number of chymase-positive cells in lung specimens of COPD patients [40,121]. Specifically, the numbers of chymase-positive cells were found to be higher in peripheral airway cells as compared to central airways. In addition, the numbers of chymase-positive cells were found to be positively correlated with FEV_1_% predicted among the COPD patients [121]. Moreover, chymase was shown to stimulate mucin production by the human bronchial epithelial cells [123]. Therefore, inhibition of chymases may be of interest in COPD treatment.

Many investigators have shown that inhibition of chymase has a profound effect on vascular remodeling, PAH, and atherosclerosis. Chymase inhibitors like BAY 1142524, RO5066852, TY-51469, JNJ-10311795, and many others have been tested in PF, atherosclerosis, and inflammation (Figure 1 and Figure 2) [83,124,125,126] but studies relating to their effect on COPD have been very limited. De Garavilla et al. provided the first evidence of the anti-inflammatory effect of chymase inhibitor JNJ-10311795 against LPS-induced airway inflammation [83]. The JNJ-10311795 inhibitor was shown to reduce inflammatory mediators within 24 h of LPS instillation. However, because of its low oral bioavailability (<1%) and low plasma half-life in rats, the aerosolized administration was postulated to be a more effective way of treating airway inflammation. In another study, JNJ-10311795 exhibited anti-inflammatory properties in a smoke-induced airway inflammation model [84]. Therefore, it will be very useful to study the anti-inflammatory effect of chymase inhibitors in COPD models to dissect the underlying mechanisms and may provide an alternative therapeutic target for COPD treatment in the near future.

## 4. The Role of MMPs and Their Inhibitors in COPD

MMPs are zinc- and calcium-dependent endopeptidases responsible for extracellular matrix remodeling [127]. There are more than 20 MMPs believed to be involved in various pathological conditions including inflammation. On the basis of substrate specificity, MMPs are classified as collagenases, gelatinases, stromelysins, elastases and membrane-bound proteinases [128]. In addition to transcriptional activation and post-transcriptional modifications, the functional activities of MMPs were also found to be regulated by the TIMPs [129].

**Figure 3:**
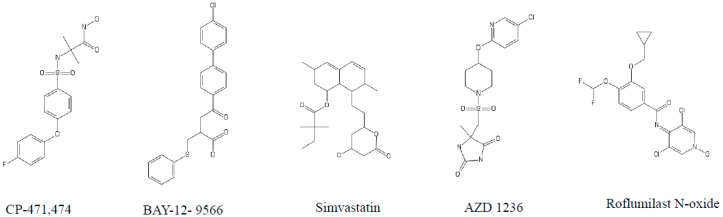
Structure of different matrix metalloprotease inhibitors tested against COPD models.

Several investigators, through both in vitro and in vivo studies, have validated the role of MMPs in emphysema pathophysiology [28,130,131,132,133]. Thus, many studies have been undertaken to study the effect of both specific and broad-spectrum MMP inhibitors for emphysema treatment. Among them, the first randomized, double-blind study was undertaken by Salmen et al. [134] who tested the efficacy of a broad spectrum MMP inhibitor, CP-471,474, in a cigarette smoke-induced emphysema model in guinea pigs (Figure 1). CP-471,474 reduced the level of MMP-1 within 2 months after smoke exposure. Moreover, it reduced the alveolar size and destruction of lung parenchyma as compared to smoke-treated guinea pigs. In another set of experiments, Pemberton et al. tested the efficacy of the inhaled MMP inhibitor, ilomastat, in a chronic smoke-exposed mouse model (Figure 1 and Figure 3). Ilomastat reduced lavage neutrophil and macrophage counts at the 6-month time point [135]. In addition, it also reduced the airspace size as compared to smoke-exposed animals alone. Ma et al. synthesized a substituted γ-Keto carboxylic acid (1j) from BAY 12-9566, a selective inhibitor of MMP-12, and tested its efficacy against a PPE-induced emphysema model (Figure 1 and Figure 3) [136]. This inhibitor reduced elastase-induced increase in lung wet weights, and morphometric analysis also showed that it protected alveolar septal walls and elastic fibres from proteolytic cleavage. Moreover, the histological data showed that it also protected the lung against hemorrhage induced by cigarette smoke exposure. On the other hand, a dual inhibitor of MMP-9/MMP-12 and AZ11557272, protected mice against an increase in small airway thickness and increases in total lung capacity, residual volume and vital capacity in smoke-exposed guinea pigs (Figure 1 and Figure 3) [137]. However, a clinical, randomized, double-blind, placebo-controlled study with selective MMP-9 and MMP-12 inhibitor, AZD1236, did not yield any significant effect in reducing symptoms associated with moderate /severe COPD [138].

Simvastatin, a lipid-lowering medication was found to effectively reduce the emphysematous changes in murine models (Figure 1 and Figure 3) [139,140]. Simvastatin reduced changes in the L_m_ of lung and lung destruction significantly in smoke-treated mice. In addition, simvastatin reduced MMP-8 and MMP-9 activity in this model (139). Further, clinical studies with salmeterol/fluticasone significantly reduced the levels of IL-8 and MMP-9 in sputum samples of treated COPD patients [141].

These studies indicate the importance of MMP inhibition in reducing emphysema in rodent models of COPD. However, few clinical studies have been performed looking directly at MMP inhibition, due to the off-target effects of some of these inhibitors. Therefore, the development of more refined and specific MMP inhibitors will be necessary for future development for the treatment of COPD.

## 5. The Role of Cysteine Proteases and Their Inhibitors in COPD

### 5.1. Caspases

Although the protease: Antiprotease imbalance theory is considered to be an important mechanism underlying emphysema development, other mechanisms may explain pathological changes associated with the development of emphysema [142,143]. The vascular theory envisages the chronic loss of both epithelium and endothelium cells of the lung due to altered programmed cell death. Aoshiba et al. showed that a single intratracheal injection of active caspase-3 into the mouse lung could induce emphysematous changes [144]. These results were further validated by Yokohori, et al. through clinical studies in patients with emphysema [145]. They showed that the percentage of alveolar wall cells undergoing apoptosis and the total number of alveolar wall cells undergoing proliferation was higher in emphysema patients compared to healthy smokers and non-smokers. Further, emphysematous lungs exhibited other signs of apoptosis such as DNA fragmentation, the presence of active caspase-3, Bad, Bax, and fragmented poly (ADP-Ribose) polymerase in lung homogenate [48,146,147]. These increases in apoptosis were mediated by a variety of inflammatory mediators including the IL-1 receptor, IL-18 receptor-α, P2X7 receptor, endothelin-1 receptor, and the NLRP3 inflammasome [47,53,148,149,150].

**Figure 4:**
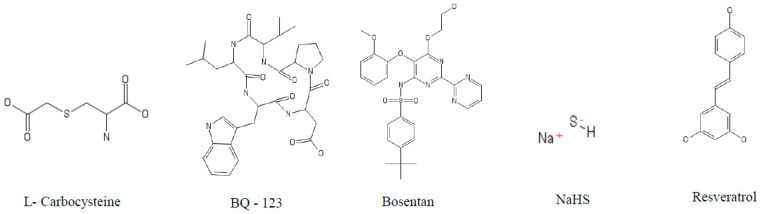
Structure of different cysteine protease inhibitors tested against COPD models.

L-Carbocysteine, a well-known mucolytic agent, was shown to inhibit hydrogen peroxide-mediated caspase-3 and caspase-9 activation through Akt phosphorylation in airway epithelial cells [151]. Moreover, carbocysteine also reduced the airspace enlargement and alveolar destruction of rat lungs exposed to cigarette smoke and lowered the mRNA expression of caspase-3 in the lung parenchyma of this model (Figure 1 and Figure 4) [152]. Administration of BQ-123 and bosentan, both endothelin-1 antagonists, for 21 days reduced smoke-induced increases in both L_m_ and destructive index in lung tissue (Figure 1). In addition, it also lowered the distribution of caspase-3 positive cells as well as caspase-3 mRNA expression in lung tissue [150]. Intraperitoneal injection of hydrogen sulfide (H_2_S) donor sodium hydrosulfide (NaHS) in a smoke-induced emphysema model inhibited smoke-induced oxidative stress, caspase-3 activation and emphysema in mouse lungs (Figure 1 and Figure 4) [153]. Moreover, it attenuated the TNF-α levels, neutrophil, and monocyte counts and decreased smoke-induced bronchial wall thickness. In an in-depth in vitro study, resveratrol was showed to protect bronchial epithelial cells from smoke-mediated apoptosis by attenuating the expression of caspase-3 and caspase-4 (Figure 1 and Figure 4) [154]. These studies provide evidence for targeting caspase-mediated apoptotic pathways in order to ameliorate emphysema development. However, the basic understanding of underlying mechanisms behind the role of apoptosis in emphysema pathogenesis is still in its infancy and further work needs to be done to translate these findings to the clinic.

### 5.2. Cathepsin S (cat S)

Cathepsin S (cat S) is an elastolytic cysteine protease with both intracellular and extracellular activities including tissue remodeling [55]. Recent studies have shown an increased level of serum cat S in COPD patients which were inversely correlated with severe airway limitation [55]. Zheng et al. showed that IFN-γ is a potent stimulator of cat S and selective inhibition of cat S attenuates the IFN-γ induced DNA damage, emphysema, and apoptosis in murine models [155]. Increased numbers of CD8+ T lymphocytes in peripheral airways was found to be associated with COPD [156,157,158]. In addition, the CD8+ cell count in the bronchial biopsies has also been found to be inversely correlated with lung function (FEV_1_) in chronic bronchitis (CB) patients [159]. More in-depth studies reveal that IFN-γ, a crucial product of CD8+ T lymphocytes, was linked with alveolar enlargement, neutrophilic inflammation and enhanced complications underlying emphysema with concomitant induction and activation of various cathepsins and MMPs [160]. Selective inhibition and genetic knockdown studies of cat S further illustrated the underlying mechanism behind IFN-γ induced emphysema pathophysiology [155].

Interestingly, secretory leukocyte protease inhibitor (SLPI), which is predominantly secreted at airway mucosal surfaces during inflammation, decreased IFN-γ induced cat S expression [161]. Geraghty et al. showed that the SLPI inhibited IFN-γ induced IκB β degradation and subsequently reduced cat S expression in macrophages (Figure 1) [161]. Although there is some evidence that cat S may play a role in COPD pathophysiology, no clinical trials directed towards this protease has been conducted so far. Moreover, studies with COPD genetic models will provide more validation to the use of anti-cat S therapy for COPD treatment.

### 5.3. Cathepsin K (cat K)

Cathepsin K (cat K), a lysosomal cysteine protease, was found to be secreted by lung epithelial cells [162]. Although the role of cat K is well known in lung fibrosis, very little is known about its potential role in COPD. Only one study has demonstrated increased expression of cat K in lung homogenates of COPD patients [54]. In addition, they showed that chronic smoke exposure significantly increased cat K expression by alveolar macrophages.

## 6. The Role of Aspartic Proteases and Their Inhibitors in COPD

### 6.1. Cathepsin D (cat D)

Cathepsin D (cat D) is an aspartyl endopeptidase primarily involved in the degradation of proteins in lysosomal compartments [163]. In addition, it plays an important role in antigen processing, cell proliferation, and activation of various bioactive protein precursors [164,165]. Moreover, cat D has also been found to be associated with emphysema [160]. Similar to the activation mechanism (described earlier), IFN-γ plays an important role in the induction and activation of cat D [166]. Moreover, an increased expression of cat D localized primarily in macrophages was observed in a smoke-exposed murine model [56]. However, the scientific knowledge in the area of cat D mediated emphysema pathogenesis is very limited to date.

### 6.2. Cathepsin E (cat E)

Cathepsin E (cat E), a major intracellular non-lysosomal aspartyl protease, plays an important role in antigen processing [167]. cat E was found to be mainly associated with different types of cancer [168,169,170]. Elevated expression of cat E is associated with airflow limitation in COPD patients [58,59] and found to be inversely correlated with FEV_1_% predicted in COPD patients. Upon deciphering the underlying mechanism behind increased expression of cat E in COPD, it was revealed that it mediates the increased expression of mitochondrial fission protein dynamin-related protein 1 and activates the caspase-dependent apoptosis pathway leading to parenchymal destruction in smoke-exposed murine models [59]. Although there is limited information available linking cat E expression and COPD pathogenesis, more in-depth mechanistic studies are required in order to understand the basic physiology behind such activity.

## 7. Conclusions

In COPD, dysregulated protease activity results in upregulation of proinflammatory mediators, increased recruitment of inflammatory cells to the lung, inactivation of important innate and antimicrobial proteins resulting in sustained inflammation and destruction of lung tissue. One way to treat such protease-mediated events in COPD is with protease inhibitor therapy. However, the translation of promising protease inhibitors from relevant in vivo models to the clinic has been disappointing thus far. Many clinical trials have focused on the ‘short-term’ benefits of protease inhibitor treatment but longer-term clinical trials may be required in order to more confidently assess the impact of inhibitor therapy. In addition, due to the presence of multiple protease activities in the COPD lung, it may be important to identify definitively whether there is a key protease or proteases central to direct tissue destruction or activation of other proteases in the diseased lung. Under these circumstances, neutralisation of one such protease with a specific protease inhibitor may lessen the overall protease burden in COPD without the need for multiple inhibitors.

## Abbreviations

A1AT	α-1 antitrypsin
Akt	Protein kinase B
Bad	Bcl-2-associated death promoter
BAL	Broncho alveolar lavage
Bax	BCL2-associated X *protein*
BbCI	*Bauhinia bauhinioide scruzipain* inhibitor
BmTI-6-D1	Kunitz-type serine protease inhibitor 6 Recombinant Protein Domain 1
CB	Chronic bronchitis
CE/CSE	Cigarette smoke extract
COPD	Chronic obstructive pulmonary disease
CXCL 12	C-X-C motif chemokine 12
CXCR1	C-X-C chemokine receptor type 1
DPP IV	Dipeptidyl peptidase IV
eNOS	Endothelial *nitric oxide synthases*
FcγRIIIb	Fcγ receptor IIIb
FEV_1_/VC	Forced expiratory volume (first second)/vital capacity.
H_2_O_2_	Hydrogen peroxide
IFN-γ	*Interferon gamma*
IL-1	Interleukin 1
IL-18	Interleukin 18
IL-1β	*Interleukin* 1 beta
IL-8	Interleukin 8
*iNOS*	*Inducible nitric oxide synthase*
L_m_	Mean linear intercept
LPS	Lipopolysaccharide
MCP-1	Monocyte chemoattractant protein 1
MMP	Matrix metalloproteinase
NaHS	Sodium hydrosulfide
NE	Neutrophil elastase
NLRP3	NACHT, LRR and PYD domains-containing protein 3
P2X7	P2X purinoceptor 7
PAH	Pulmonary arterial hypertension
PF	Pulmonary fibrosis
PPE	Porcine pancreatic elastase
PR3	Proteinase 3
rBbKI	recombinant *Bauhinia bauhinioides* Kallikrein proteinase Inhibitor
RLV	Relative lung volumes
RVH	Right ventricular hypertrophy
SLPI	Secretory leukocyte protease inhibitor
Smad	Mothers against decapentaplegic homolog transcription factor
TGF-β	Transforming growth factor beta 1
TNFα	Tumor necrosis factor-alpha
V_L_	Lung volumes
WT-elafin	Wild type elafin
